# VCP/p97 mediates nuclear targeting of non-ER-imported prion protein to maintain proteostasis

**DOI:** 10.26508/lsa.202302456

**Published:** 2024-04-03

**Authors:** Papiya Banik, Koustav Ray, Janine Kamps, Qi-Yin Chen, Hendrik Luesch, Konstanze F Winklhofer, Jörg Tatzelt

**Affiliations:** 1 Department Biochemistry of Neurodegenerative Diseases, Institute of Biochemistry and Pathobiochemistry, Ruhr University Bochum , Bochum, Germany; 2 Department Molecular Cell Biology, Institute of Biochemistry and Pathobiochemistry, Ruhr University Bochum , Bochum, Germany; 3 Cluster of Excellence RESOLV, Bochum, Germany; 4 Department of Medicinal Chemistry and Center for Natural Products, Drug Discovery and Development (CNPD3), University of Florida , Gainesville, FL, USA

## Abstract

Mistargeting of secretory proteins in the cytosol can trigger their aggregation and subsequent proteostasis decline. We have identified a VCP/p97-dependent pathway that directs non-ER-imported prion protein (PrP) into the nucleus to prevent the formation of toxic aggregates in the cytosol. Upon impaired translocation into the ER, PrP interacts with VCP/p97, which facilitates nuclear import mediated by importin-ß. Notably, the cytosolic interaction of PrP with VCP/p97 and its nuclear import are independent of ubiquitination. In vitro experiments revealed that VCP/p97 binds non-ubiquitinated PrP and prevents its aggregation. Inhibiting binding of PrP to VCP/p97, or transient proteotoxic stress, promotes the formation of self-perpetuating and partially proteinase resistant PrP aggregates in the cytosol, which compromised cellular proteostasis and disrupted further nuclear targeting of PrP. In the nucleus, RNAs keep PrP in a soluble and non-toxic conformation. Our study revealed a novel ubiquitin-independent role of VCP/p97 in the nuclear targeting of non-imported secretory proteins and highlights the impact of the chemical milieu in triggering protein misfolding.

## Abstract (precis)

The study shows a novel ubiquitin-independent role of VCP/p97 in the nuclear targeting of non-imported secretory proteins to prevent formation of toxic protein aggregates in the cytosol.

## Introduction

To maintain cellular protein homeostasis and to preclude toxic effects of aberrant protein conformers, components of the proteostasis network ensure proper protein folding and recognition and degradation of misfolded and non-functional proteins. The accumulation of protein aggregates in various neurodegenerative diseases suggests that an overload of quality control pathways and/or a decline in their efficiencies play a crucial role in the pathogenesis of these diseases (Taipale et al, 2010; Hartl, 2017; Schopf et al, 2017; Mogk et al, 2018). Another determinant of protein folding, misfolding, and aggregation is the distinct chemical milieu of the cellular compartments. For example, the folding of membrane proteins and secretory proteins, which often contain disulfide bonds, is critically dependent on the specialized environment in the endoplasmic reticulum (Jessop et al, 2004; Brodsky & Skach, 2011; Braakman & Hebert, 2013). As a consequence, mislocalization of these proteins in the cytosol can trigger the formation of toxic protein aggregates (Juszkiewicz & Hegde, 2018; Hegde & Zavodszky, 2019).

The major quality control pathway for misfolded secretory proteins is endoplasmic reticulum (ER)-associated degradation (ERAD). This pathway involves recognition of non-native polypeptides in the ER lumen by ER-resident proteins and retrograde transport to the cytosol for proteasomal degradation (rev. in Ellgaard and Helenius [2003], Meusser et al [2005], Nakatsukasa and Brodsky [2008], Krshnan et al [2022], and Christianson et al [2023]). In the cytosol, the AAA (ATPases with diverse cellular activities) protein VCP/p97 has a pivotal role in ERAD. After ubiquitination of ERAD substrates on the cytosolic side, their complete retro-translocation and subsequent targeting to cytosolic proteasomes for degradation are coordinated by VCP/p97 (Ye et al, 2003; Neuber et al, 2005; Schuberth & Buchberger, 2005). In addition to ubiquitination, several adaptor proteins/cofactors have been identified that provide substrate and targeting specificity (Buchberger et al, 2015; Meyer & van den Boom, 2023; Meyer & Weihl, 2014; Stach & Freemont, 2017).

A prominent example of a secretory protein with neurotoxic potential is the mammalian prion protein (PrP). Prion diseases are invariably fatal neurodegenerative disorders in humans and animals caused by the conformational transition of the cellular PrP (PrP^C^) into pathogenic conformers, denoted scrapie prion protein (PrP^Sc^), that are infectious and neurotoxic (Prusiner, 1998; Aguzzi & Polymenidou, 2004; Wadsworth & Collinge, 2011). Interestingly, WT PrP^C^ forms cytosolic aggregates after transient proteasomal inhibition, which is accompanied by decreased cellular viability (Ma & Lindquist, 2001, 2002; Yedidia et al, 2001; Ma et al, 2002; Drisaldi et al, 2003; Heller et al, 2003; Rane et al, 2004; Kristiansen et al, 2005; Rambold et al, 2006; Kristiansen et al, 2007; Norstrom et al, 2007; Chakrabarti & Hegde, 2009; Deriziotis et al, 2011). Initially, it was assumed that cytosolic PrP is generated through the ERAD pathway. However, work from the Hegde group revealed that 10–20% of newly synthesized PrP is not even imported into the ER because of an inefficient ER signal peptide (Kim et al, 2001; Rutkowski et al, 2001; Drisaldi et al, 2003; Heller et al, 2003; Heske et al, 2004; Rane et al, 2004; Hegde & Bernstein, 2006; Rambold et al, 2006; Miesbauer et al, 2009).

The concept that non-ER-imported PrP is subjected to proteasomal degradation in the cytosol has mainly been inferred from the cytosolic accumulation of PrP after proteasomal inhibition. Here, we re-evaluated this concept by using novel cell culture and in vitro approaches. Remarkably, we found that PrP does interact with VCP/p97 after an aborted ER import; however, it is not targeted to cytosolic proteasomes but to the nucleus. Moreover, the interaction of PrP with VCP/p97 and the nuclear targeting of PrP are independent of ubiquitination.

## Results

### Non-ER-imported PrP is targeted to the nucleus

**Figure 1.**
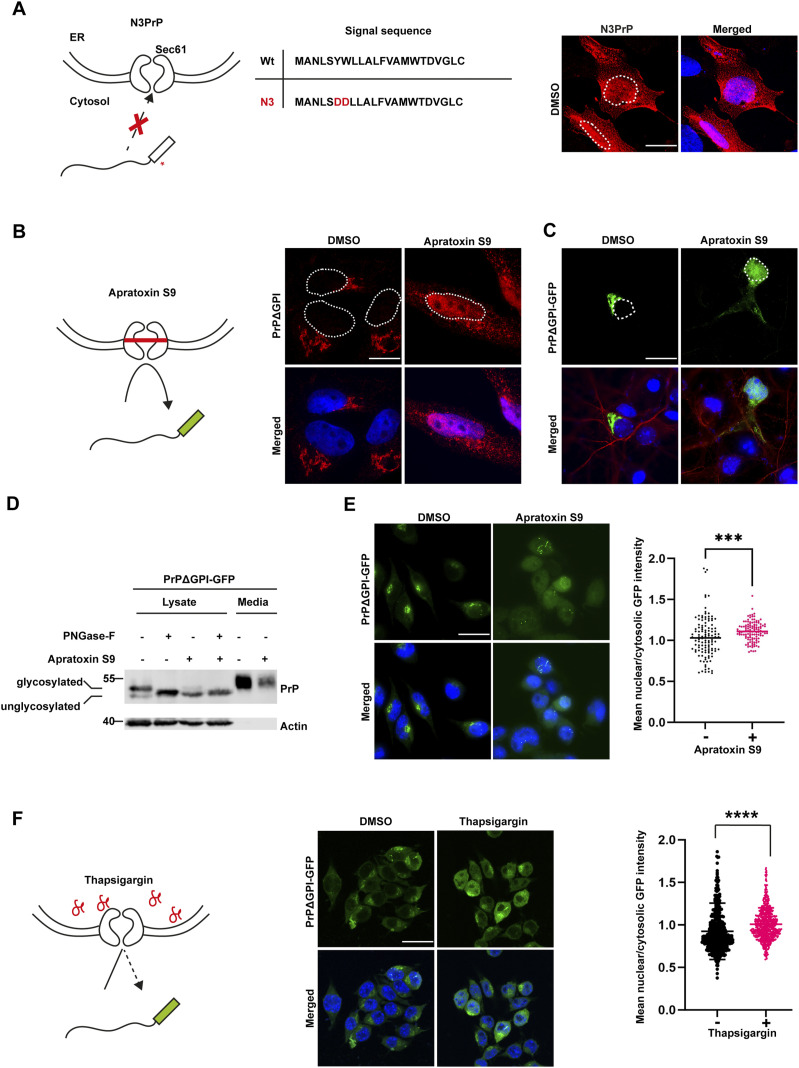
After failed import into the ER, PrP is targeted to the nucleus. **(A, B, C, D, E, F)** Nuclear targeting of non-ER-imported PrP in different cell lines and under different conditions. **(A)** N3PrP has mutations in the signal peptide preventing its interaction with the signal recognition particle and/or gating and thereby ER import. SH-SY5Y cells transiently expressing N3PrP were stained with antibodies against PrP and analyzed by SR-SIM. White dotted lines indicate the boundaries of the nuclei. Nuclei were stained with DAPI (merged). **(B, C, D, E)** Apratoxin S9 inhibits co-translational translocation of secretory proteins through the Sec61 translocon. **(B)** SH-SY5Y cells transiently expressing PrPΔGPI were treated with Apratoxin S9 (100 nM) for 4 h and fixed, stained with antibodies against PrP and analyzed by SR-SIM. White dotted lines indicate the boundaries of the nuclei. Nuclei were stained with DAPI (merged). **(C)** Primary cortical mouse neurons at DIV 6 were transfected with PrPΔGPI-GFP. After 48 h post-transfection, the cells were treated for 4 h with either DMSO or Apratoxin S9 (100 nM), fixed, stained with antibodies against MAP2 and analyzed by SR-SIM. White dotted lines indicate boundaries of the nuclei. Nuclei were stained with DAPI (merged). **(D)** HEK293T cells were transiently transfected with PrPΔGPI-GFP. 24 h post-transfection, cells were treated for 4 h with either DMSO or Apratoxin S9 (100 nM) in fresh media. Cells were lysed and digested with PNGase-F or left untreated. Cell lysates and conditioned media were analyzed by immunoblotting using antibodies against PrP and β-Actin (loading control). Glycosylated and unglycosylated PrP species are indicated. **(E)** N2a cells stably expressing PrPΔGPI-GFP were plated and treated for 4 h with either DMSO or Apratoxin S9 (100 nM), fixed and GFP fluorescence was analyzed by SR-SIM (left panel). Nuclei were stained with DAPI (merged). Using CellProfiler software, the ratio of nuclear to cytosolic mean GFP intensity was quantified. The indicated line is the mean of the data set and was analyzed by two-tailed Mann-Whitney test at 95% confidence interval, ***P = 0.0005. At least 50 cells were analyzed per biological replicate (n = 3) (right panel). **(F)** N2a cells stably expressing PrPΔGPI-GFP were treated for 4 h with either DMSO or Thapsigargin (5 μM) to induce ER stress. The cells were fixed and GFP fluorescence was analyzed by SR-SIM (left panel). Nuclei were stained with DAPI (merged). Using CellProfiler software, the ratio of nuclear to cytosolic mean GFP intensity was quantified. The indicated line is the mean of the data set and was analyzed by two-tailed Mann-Whitney test at 95% confidence interval, ****P = 0.0001. At least 50 cells were analyzed per biological replicate (n = 3) (right panel). Scale bar, 10 μm.

**Figure S1.**
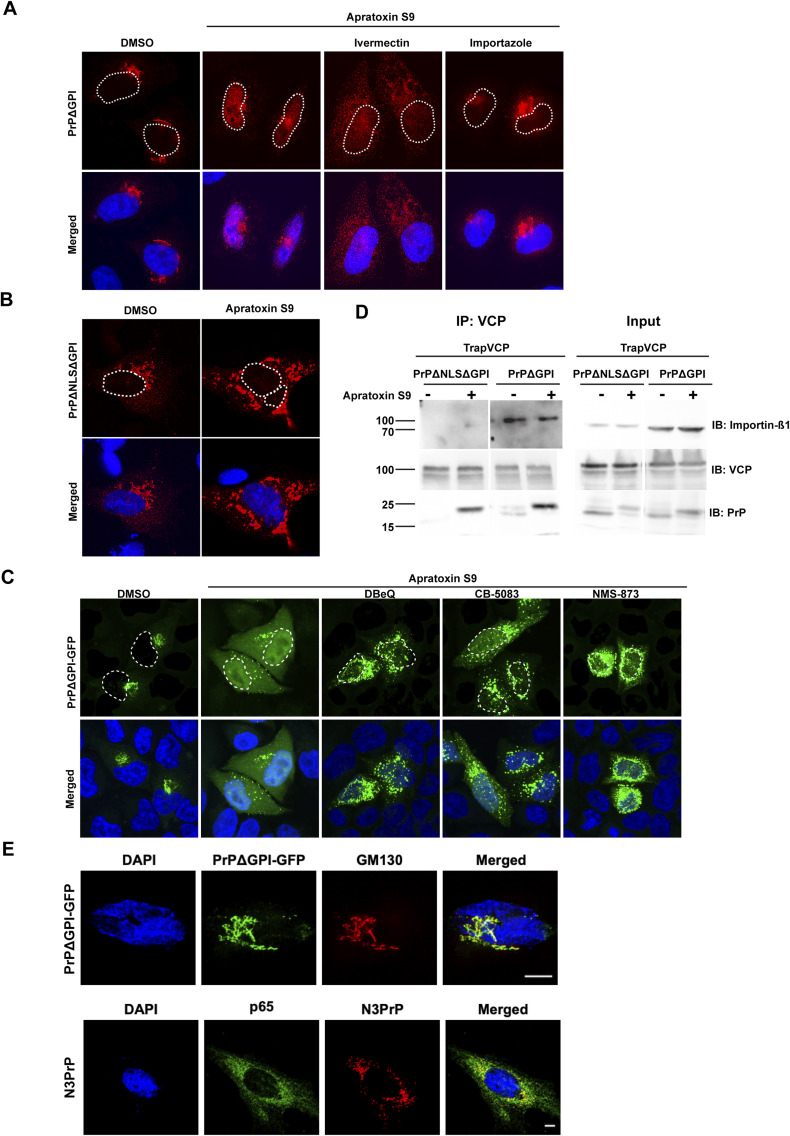
A cryptic NLS in PrP is required for nuclear import of PrP. **(A)** Pharmacological inhibition of importins decreases nuclear import of non-ER-imported PrP. HeLa cells were transiently transfected with PrPΔGPI. 24 h post-transfection cells were treated for 3 h with Apratoxin S9 (100 nM), Apratoxin S9 with Ivermectin (50 μM) or Importazole (50 μM). Note that importin inhibitors were added 1 h before Apratoxin S9. Cells were fixed, stained with antibodies against PrP and analyzed by SR-SIM. Nuclei were stained with DAPI (merged). White dotted lines indicate boundaries of the nuclei. **(B)** Mutating a cryptic NLS in PrP decrease nuclear import. SH-SY5Y cells transiently expressing PrPΔNLSΔGPI were fixed, stained with antibodies against PrP and analyzed by SR-SIM. Nuclei were stained with DAPI (merged). **(C)** Pharmacological inhibition of VCP/p97 decreases nuclear import and increases cytosolic accumulation of non-ER-imported PrP. HeLa cells transiently transfected with PrPΔGPI-GFP were treated for 3 h with Apratoxin S9 (100 nM), or with Apratoxin S9 together with the VCP/p97 inhibitors DBeQ (10 μM), or NMS-873 (10 μM) or CB-5083 (10 μM). Please note that the VCP inhibitors were added 1 h before Apratoxin S9. The cells were fixed and GFP fluorescence was analyzed by SR-SIM. Nuclei were stained with DAPI (merged). White dotted lines indicate boundaries of the nuclei. **(D)** Non-ER-imported PrP forms a ternary complex with VCP/p97 and importin-β1. HEK293T cells co-expressing Strep-tagged VCP/p97 and PrPΔGPI or PrPΔNLSΔGPI were subjected to an immunoprecipitation under native conditions with Streptactin magnetic beads. The immunoblots were tested for antibodies against PrP, VCP/p97 and endogenous importin-β1. **(E)** PrPΔGPI-GFP is in the secretory pathway, whereas N3PrP is in the cytosol. SH-SY5Y cells transiently expressing PrPΔGPI-GFP or N3PrP were fixed, stained with antibodies against PrP, a marker for the Golgi (GM130), or for the cytosol (p65) and analyzed by SR-SIM. Nuclei were stained with DAPI (merged).

To specifically study cytosolic quality control pathways that operate on the fraction of secretory proteins that were not translocated through the Sec61 translocon into the ER, we followed up on three independent approaches to generate non-imported prion proteins (Fig 1A, B, and F). First, we studied N3PrP, a full-length PrP variant equipped with an N-terminal ER signal peptide and a C-terminal GPI signal sequence that is not imported into the ER after synthesis because of a mutated ER signal peptide. The mutations prevent binding of the PrP nascent chain to the SRP (signal recognition particle) and/or gating and thereby ER import (Kim et al, 2002). Interestingly, N3PrP was not only present in the cytosol of human neuroblastoma SH-SY5Y cells but also in the nucleus (Fig 1A). Second, to study the fate of PrP under conditions of blocked ER import, we reversibly inhibited co-translational translocation of secretory proteins through the Sec61 translocon by Apratoxin S9, which is the most potent synthetic analogue of the cyclic depsipeptide natural products of the Apratoxin class (Liu et al, 2009; Chen et al, 2014; Itskanov et al, 2023). We expressed PrP lacking the C-terminal GPI-anchor signal sequence (PrPΔGPI) since the ER import of PrPΔGPI is comparable to that of GPI-anchored PrP, but PrPΔGPI is secreted (Winklhofer et al, 2003; Rambold et al, 2006; Campana et al, 2007). Thus, by using this mutant we can make sure that cytosolic localization of PrPΔGPI is a consequence of its impaired ER import and not of PrP misfolding in the ER lumen (Satpute-Krishnan et al, 2014), nor endocytosis of PrP from the plasma membrane (Sunyach et al, 2003). Fluorescence microscopy indicated the localization of PrPΔGPI within the secretory pathway of control cells, as expected for a secreted protein (Figs 1B and S1C). In cells treated with Apratoxin S9 for 4 h, PrPΔGPI was mostly detected in the nucleus (Fig 1B). To facilitate the microscopic analysis, we equipped PrPΔGPI with a C-terminal GFP and imaged mouse primary cortical neurons transiently expressing PrPΔGPI-GFP +/− Apratoxin S9 treatment to ensure that the transport of non-ER-imported PrP from the cytosol to the nucleus is not a specific feature of established cell lines. In line with our findings in established cell lines, non-ER-imported PrP was targeted to the nucleus in primary neurons (Fig 1C). Next, we generated mouse neuroblastoma N2a cell lines stably expressing PrPΔGPI-GFP. The activity of Apratoxin S9 to potently inhibit ER import was validated by immunoblotting of cell lysates. The upper band, representing N-linked glycosylated PrPΔGPI-GFP within the secretory pathway, was reduced remarkably in cells exposed to Apratoxin S9 for 4 h. In addition, the amount of PrPΔGPI-GFP in conditioned media decreased under these conditions (Fig 1D). Fluorescence microscopy analysis confirmed that similarly to PrPΔGPI, PrPΔGPI-GFP was transported from the cytosol to the nucleus after inhibiting ER import by Apratoxin S9 (Fig 1E).

Previous work revealed that ER stress results in an overall reduced rate of ER import of secretory proteins (Kang et al, 2006; Oyadomari et al, 2006; Hegde & Kang, 2008). Specifically, PrP has been shown to be co-translocationally targeted to proteasomal degradation during acute ER stress (Kang et al, 2006; Orsi et al, 2006). However, in these studies, the stabilization of PrP after proteasomal inhibition was only shown by Western blotting without addressing the cellular compartment of PrP accumulation. To assess the cellular localization of non-ER-imported PrP in a third approach, we made use of ER stress and analyzed transiently transfected cells treated for 4 h with Thapsigargin, a non-competitive inhibitor of the sarco/endoplasmic reticulum Ca^2+^ ATPase (Fig 1F). Fluorescence microscopy revealed that a fraction of PrPΔGPI-GFP was not imported into the ER and targeted to the nucleus in Thapsigargin-treated cells (Fig 1F, middle and right panels). In sum, our three-pronged approach revealed that non-ER-imported PrP, because of inefficient or impaired ER import during stress, is targeted at the nucleus.

### VCP/p97 and importin-ß are required for nuclear targeting of PrP

**Figure 2.**
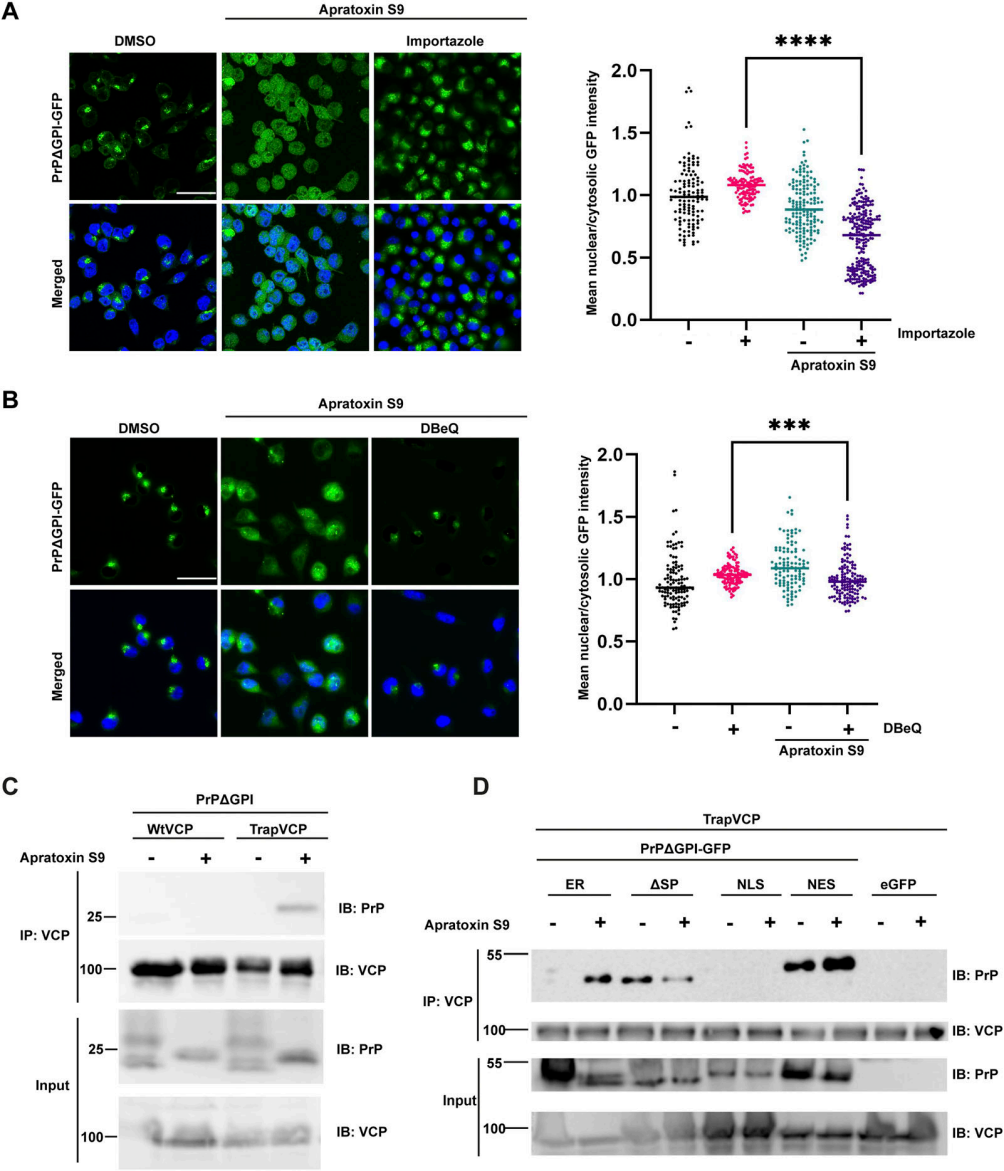
Nuclear import of PrP is dependent on Importin-β and VCP/p97. **(A)** N2a cells stably expressing PrPΔGPI-GFP were treated for 4 h with Apratoxin S9 (100 nM), Importazole (50 μM), or with both. The cells were fixed and GFP fluorescence was analyzed by SR-SIM. Nuclei were stained with DAPI (merged). Using CellProfiler software, the ratio of nuclear to cytosolic mean GFP intensity was quantified. The indicated line is the mean of the data set and was analyzed by Kruskal-Wallis test followed by Dunn’s multiple comparison test at 95% confidence interval, ****P = 0.0001. At least 50 cells were analyzed per biological replicate (n = 3). **(B)** N2a cells stably expressing PrPΔGPI-GFP were treated for 3 h with Apratoxin S9 (100 nM), or with Apratoxin S9 together with DBeQ (10 μM). Please note that DBeQ was added to the cell culture media 1 h before Apratoxin S9. The cells were fixed and GFP fluorescence was analyzed by SR-SIM. Nuclei were stained with DAPI (merged). Using CellProfiler software, the ratio of nuclear to cytosolic mean GFP intensity was quantified. The indicated line is the mean of the data set and was analyzed by Kruskal-Wallis test followed by Dunn’s multiple comparison test at 95% confidence interval, ***P = 0.0005. At least 50 cells were analyzed per biological replicate (n = 3). **(C)** VCP/p97 interacts with non-ER-imported PrP. HEK293T cells were co-transfected with PrPΔGPI and Strep-tagged WtVCP or E578QVCP (TrapVCP). 24 h post-transfection, cells were treated with DMSO or Apratoxin S9 (100 nM) for 4 h, lysed and subjected to immunoprecipitation under native conditions with Streptactin magnetic beads. The immunoblots were tested for antibodies against PrP (3F4) and VCP. **(D)** VCP/p97 interacts only with cytosolic PrP. HEK293T cells were co-transfected with Strep-tagged E578QVCP (TrapVCP) and PrPΔGPI-GFP either without an ER signal peptide (ΔSP) or containing an NLS or NES instead of the ER signal peptide. **(C)** 24 h post-transfection, cells were treated with DMSO or Apratoxin S9 (100 nM) for 4 h and analyzed as described in (C). Scale bar, 10 μm.

The preceding findings raised the question of the mechanism underlying the transport of cytosolic PrP into the nucleus. We first investigated whether the nuclear import of PrP is an active process mediated by nuclear transporters. Cells transiently expressing PrPΔGPI were treated with Apratoxin S9 in combination with different importin inhibitors specific for either importin-α or importin-ß. In the presence of Importazole, an inhibitor of importin-ß, PrPΔGPI accumulated in the perinuclear region (Figs 2A and S1A). The nuclear translocation of PrPΔGPI was minimally affected by Ivermectin, which inhibits importin-α (Fig S1A). A quantitative analysis in cells stably expressing PrPΔGPI-GFP confirmed that in the presence of Importazole, the nuclear import of PrPΔGPI-GFP in Apratoxin S9-treated cells was significantly impaired (Fig 2A). Our findings are consistent with the finding that the N-terminal domain of PrP contains a cryptic NLS (Gu et al, 2003). Indeed, mutating this NLS significantly decreased nuclear localization of PrPΔNLSΔGPI in Apratoxin S9-treated cells (Fig S1B).

In the ERAD pathway, VCP/p97 is required for the retrograde translocation of misfolded secretory proteins from the ER and their targeting to cytosolic proteasomes (Ye et al, 2003; Neuber et al, 2005; Schuberth & Buchberger, 2005). To study whether VCP/p97 is involved in nuclear targeting of PrP after an aborted ER import, we treated cells transiently expressing PrPΔGPI-GFP with Apratoxin S9 and VCP/p97 inhibitors. Inhibiting VCP/p97 by three different inhibitors, DBeQ, CB-5083, or NMS-873, decreased the nuclear localization of PrP and increased the fraction of PrP in the cytosol (Fig S1C). A quantitative analysis using the stably PrPΔGPI-GFP-expressing cell line verified that nuclear targeting of non-ER-imported PrP in DBeQ-treated cells was significantly reduced (Fig 2B). To analyze the role of VCP/p97 in more detail, we immunoprecipitated VCP/p97 under non-denaturing conditions to maintain protein-protein interactions and then analyzed co-purifying proteins for the presence of PrPΔGPI by Western blotting. Since the interaction of VCP/p97 with its client proteins is only transient, we included TrapVCP in our experiments, which is a VCP/p97 mutant (E578Q) that does not release clients after binding (Hulsmann et al, 2018). Immunoblots of the input lysates confirmed that Apratoxin S9 efficiently blocked ER import. The upper band of PrPΔGPI, representing glycosylated species in the secretory pathway, was absent in lysates prepared from Apratoxin S9-treated cells (Fig 2C). An interaction of PrPΔGPI with WT VCP/p97 could not be detected by this approach, neither in control nor in Apratoxin S9-treated cells. However, PrPΔGPI co-purified with TrapVCP in Apratoxin S9-treated cells (Fig 2C). Of note, TrapVCP did not interact with PrP in control cells without Apratoxin S9, revealing that the interaction was specific for non-ER-imported PrP. Moreover, VCP/p97 interacted specifically with cytosolic PrP and not with nuclear PrP (Fig 2D). We were also able to detect a ternary complex of VCP/p97, importin-β, and PrP, suggesting a hand-over mechanism of PrP from VCP/p97 to importin-β. Mutating the cryptic NLS of PrP abolished recruitment of importin-β to the complex (Fig S1D). Taken together, nuclear targeting of cytosolic PrP after a failed ER import depends not only on importin-ß but also on VCP/p97.

### The cytosolic interaction of PrP with VCP/p97 and PrP nuclear targeting are independent of ubiquitination

**Figure 3.**
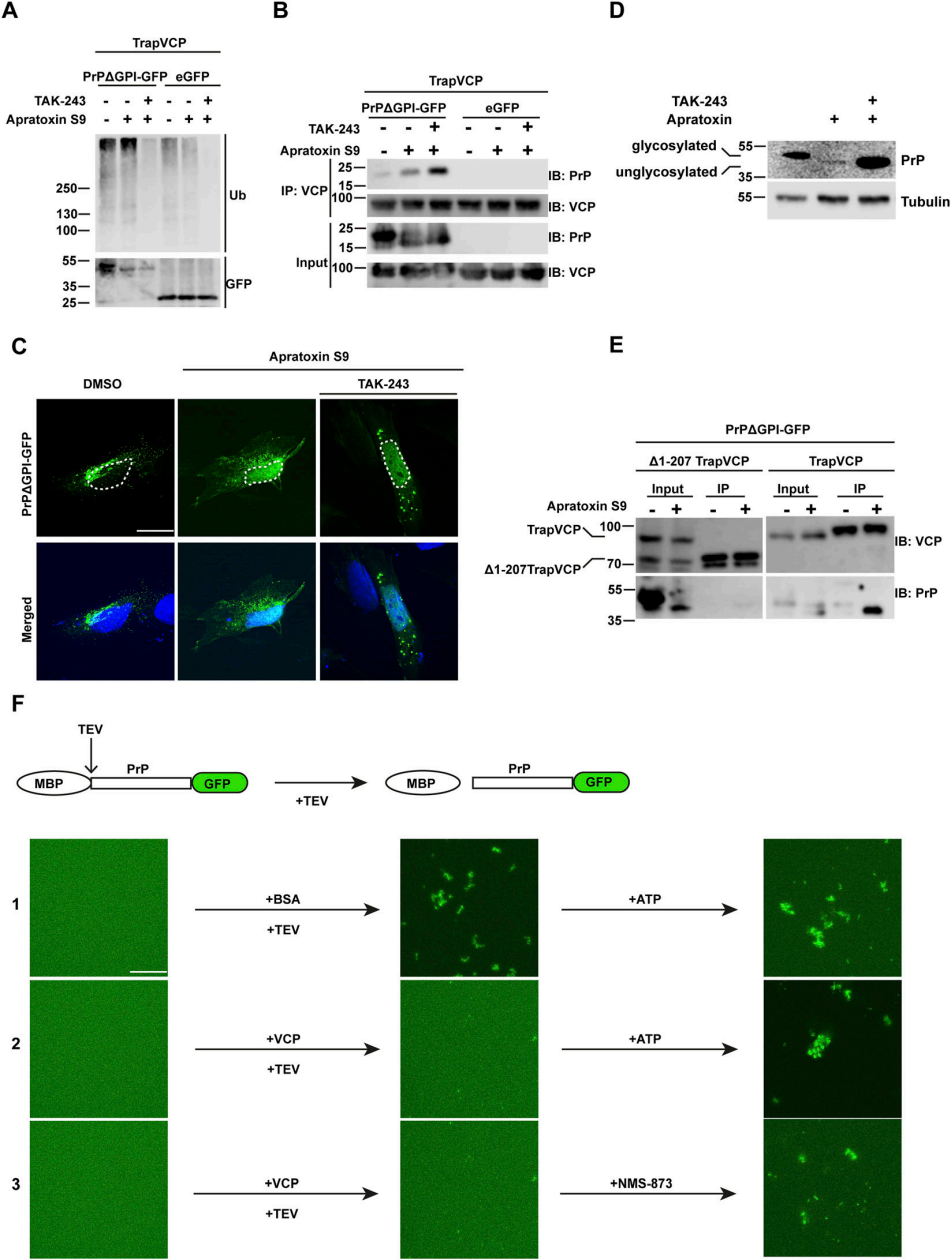
Ubiquitination of non-imported PrP is dispensable for its interaction with VCP/p97 and nuclear targeting but is required for proteasomal degradation. **(A, B)** VCP/p97 interacts with non-ER-imported PrP independent of ubiquitination. HEK293T cells were co-transfected with Strep-tagged E578QVCP (TrapVCP) and PrPΔGPI-GFP, or eGFP. 24 h post-transfection, cells were treated for 3 h with Apratoxin S9 (3 h, 100 nM) and/or TAK-243 (4 h, 1 μM) an inhibitor of ubiquitin-activating enzymes as indicated. Note that the ubiquitin-activating enzyme inhibitor was added 1 h before Apratoxin S9. **(A)** Cell lysates were analyzed by immunoblotting using antibodies against ubiquitin (P4D1) and GFP. **(B)** Cell lysates subjected to immunoprecipitation under native conditions using Streptactin magnetic beads. Precipitated proteins were then detected by immunoblotting using antibodies against PrP and VCP/p97. **(C)** Nuclear import of PrP is independent of ubiquitination. SH-SY5Y cells were transiently transfected with PrPΔGPI-GFP. 24 h post-transfection cells were treated with Apratoxin S9 (3 h, 100 nM), or Apratoxin S9 (3 h, 100 nM) and TAK-243 (4 h, 1 μM). Note that TAK-243 was added 1 h before Apratoxin S9. The cells were fixed and GFP fluorescence was analyzed by SR-SIM. White dotted lines indicate boundaries of the nuclei. Nuclei were stained with DAPI (Merged). **(D)** Ubiquitination is required for proteasomal degradation of PrP. HEK293T cells were transiently transfected with PrPΔGPI-GFP. 24 h post-transfection cells were treated with Apratoxin S9 (3 h, 100 nM), or Apratoxin S9 (3 h, 100 nM) and TAK-243 (4 h, 1 μM). Note that TAK-243 was added 1 h before Apratoxin S9. The cells were lysed and analyzed by immunoblotting using antibodies against PrP and β-Actin (loading control). Glycosylated and unglycosylated PrP species are indicated. **(E)** The flexible N-terminal tail of VCP/p97 is required for an interaction with non-ER-imported PrP. HEK293T cells were co-transfected with PrPΔGPI-GFP and Strep-tagged E578QVCP (TrapVCP) or Strep-tagged E578QVCP with deletion of aa 1–207 (Δ1-207 TrapVCP). 24 h post-transfection, cells were treated with DMSO or Apratoxin S9 (100 nM) for 4 h, lysed and subjected to immunoprecipitation under native conditions with Streptactin magnetic beads. The immunoblots were tested for antibodies against PrP (3F4) and VCP. **(F)** VCP/p97 prevents a conformational transition of soluble PrP into aggregates in vitro. Scheme of the experimental approach. MBP-PrP-GFP was expressed in *E. coli* and purified as a soluble protein. The N-terminal MBP-tag was removed by tobacco etch virus (TEV) protease to induce phase separation (top panel). (Row 1–3) 5 μM MBP-PrP-GFP was incubated in the presence of TEV protease for 1 h at RT in aggregation buffer (10 mM Tris–pH 7.4, 150 mM NaCl). To analyze aggregation of PrP-GFP fluorescence imaging data were recorded by laser scanning microscopy using Z-stack and processed with maximum intensity projection. (1) BSA (5 μM) was added together with TEV. After 1 h the reaction was incubated for an additional 1 h in the presence of 2 mM ATP. (2) Recombinant VCP/p97-GST (5 μM) was added together with TEV. After 1 h the reaction was incubated for an additional 1 h in the presence of 2 mM ATP. (3) Recombinant VCP/p97-GST (5 μM) was added together with TEV. After 1 h the reaction was incubated for an additional 1 h in the presence of the allosteric VCP/p97 inhibitor NMS-873 (10 μM). Scale bar, 10 μm.

**Figure S2.**
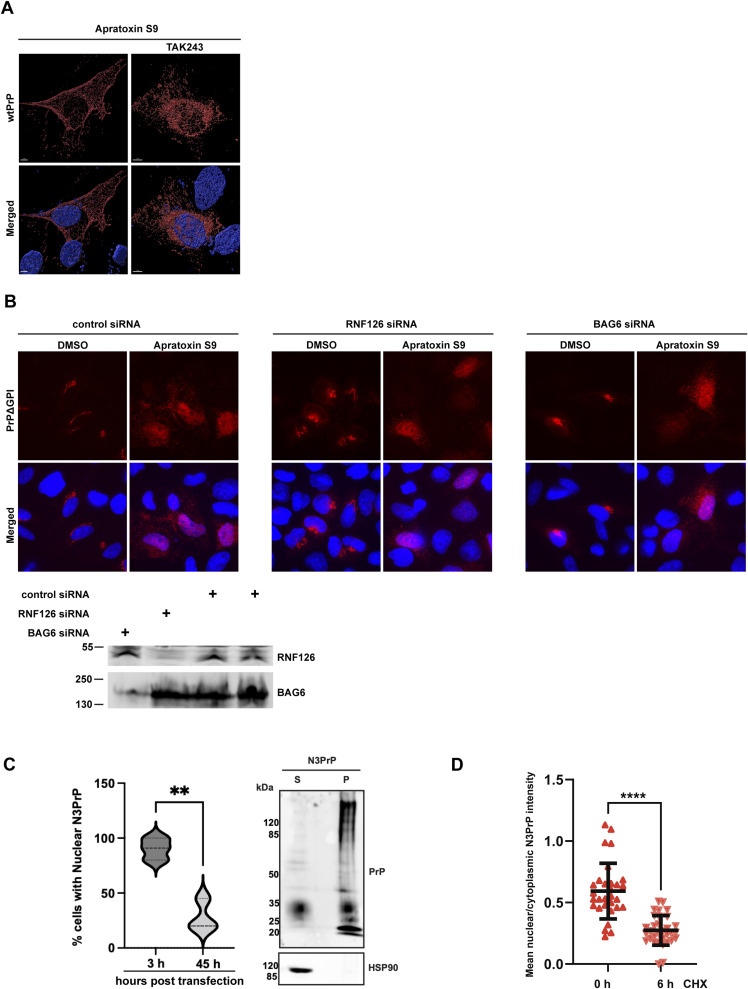
Proteasomal degradation of non-ER-imported PrP occurs mainly in the nucleus. **(A)** After impaired ER import WT PrP is targeted to the nucleus and degraded by the proteasome. SH-SY5Y cells transiently expressing WT, GPI-anchored PrP were treated with E1 enzyme inhibitor TAK-243 (1 μM) and Apratoxin S9 (100 nM) for 4 h, fixed, stained with antibody against PrP and analyzed by SR-SIM. Nuclei were stained with DAPI (merged). Shown is a volumetric 3D reconstruction generated with the IMARIS software. **(B)** Transient knockdown of BAG6 or RNF126 does not interfere with nuclear import of PrP. HeLa cells were transiently transfected with PrPΔGPI and siRNA against RNF126, or BAG6. 24 h post-transfection cells were treated for 4 h with Apratoxin S9 (100 nM). The cells were fixed, stained with antibody against PrP and analyzed by SR-SIM. Nuclei were stained with DAPI (merged). Knockdown efficiencies were analyzed by immunoblotting of cell lysates using antibodies against RNF126 or BAG6 (lower panel). **(C)** A decrease in nuclear N3PrP is accompanied by an accumulation of detergent-insoluble N3PrP in the cytosol. SH-SY5Y cells were transfected with N3PrP, the cells were fixed, stained with antibody against PrP, and analyzed by SR-SIM at indicated time points post-transfection. Number of cells with nuclear PrP was counted and plotted at different time points. At least 40 cells were analyzed per biological replicate (n = 3). The violin plot represents individual data point and was analyzed by an unpaired *t* test at 95% confidence interval, **P = 0.005 (left panel). In a parallel experiment, cells expressing N3PrP were collected 45 h post-transfection, lysed, and separated into a detergent-soluble (S) and -insoluble (P) fraction. The fractions were analyzed by Western blotting using an antibody against PrP. HSP90 was analyzed as a control (right panel). **(D)** Nuclear PrP is degraded faster than cytosolic PrP. SH-SY5Y cells were transiently transfected with N3PrP. 24 h post-transfection cells were treated for 6 h with 50 μg/ml cycloheximide to inhibit translation. Cells were then fixed, stained with antibodies against PrP and analyzed by laser scanning microscopy. Using Image J software, the ratio of nuclear to cytosolic mean GFP intensity was quantified. Error bars represent mean ± s.d. and was analyzed by Welch’s *t* test followed 95% confidence interval, ****P < 0.0001. At least 30 cells were analyzed in biological replicates of n = 3.

Ubiquitination of client proteins has been shown to mediate their interaction with VCP/p97. However, recent research has revealed that VCP is capable of interacting with substrates directly, independent of ubiquitination or adaptor proteins (van den Boom et al, 2023; Weith et al, 2018). To explore the role of ubiquitination in the interaction of non-ER-imported PrP with VCP/p97 and its nuclear targeting, cells transiently expressing PrPΔGPI-GFP were treated with Apratoxin S9 in combination with an inhibitor of the E1 ubiquitin-activating enzyme (TAK-243). Immunoblotting of whole cell lysates showed that TAK-243 efficiently inhibited ubiquitination of proteins (Fig 3A). In parallel, we immunoprecipitated TrapVCP and probed the immunopellet for PrP. Notably, non-ER-imported PrPΔGPI-GFP co-purified with TrapVCP also in TAK-243-treated cells, indicating that the PrP-VCP/p97 interaction was independent of ubiquitination (Fig 3B). Likewise, ubiquitination was not required for nuclear targeting since PrPΔGPI-GFP localized to the nucleus in cells treated with Apratoxin S9 and TAK-243 (Fig 3C). However, in the presence of TAK-243, non-ER-imported PrP was stabilized, indicating that ubiquitination was required for proteasomal degradation of PrP (Fig 3D). Importantly, we also observed nuclear accumulation of WT PrP in cells treated with Apratoxin S9 and TAK-243. This demonstrates that our findings are also relevant to untagged, GPI-anchored PrP^C^ (Fig S2A).

It was shown recently that the flexible N-terminal tail of VCP/p97 mediates the interaction with non-ubiquitinated substrates (van den Boom et al, 2023). Consequently, we analyzed whether the N-terminal tail of VCP/p97 is required for an interaction with non-ER-imported PrP. To this end, we immunoprecipitated a TrapVCP variant lacking the flexible N-terminal domain (Δ1-207) under non-denaturing conditions to maintain protein-protein interactions and then analyzed co-purifying proteins for the presence of PrPΔGPI-GFP by Western blotting. Similar to PrPΔGPI (Fig 2C), full-length TrapVCP interacted with PrPΔGPI-GFP in Apratoxin S9-treated cells. Remarkably, PrP did not co-precipitate with Δ1-207TrapVCP, suggesting that the interaction with non-ER-imported PrP is mediated by the flexible N-terminal tail of VCP/p97 (Fig 3E).

BAG6 and RNF126 have been described previously to interact with PrP after its aborted ER import (Hessa et al, 2011; Rodrigo-Brenni et al, 2014). To test whether these factors are implicated in nuclear targeting of non-ER-imported PrP, we down-regulated their expression by siRNAs in Apratoxin S9-treated cells transiently expressing PrPΔGPI. Silencing of neither BAG6 nor RNF126 impeded nuclear targeting of PrP (Fig S2B). As a conclusion, the interaction of VCP/p97 with PrP after its failed ER import is independent of ubiquitination but requires the flexible N-terminal tail of VCP/p97. Ubiquitination is also dispensable for nuclear targeting of non-ER-imported PrP; however, ubiquitination of PrP is required for proteasomal degradation.

### VCP/p97 prevents aggregation of PrP in vitro

**Figure S3.**
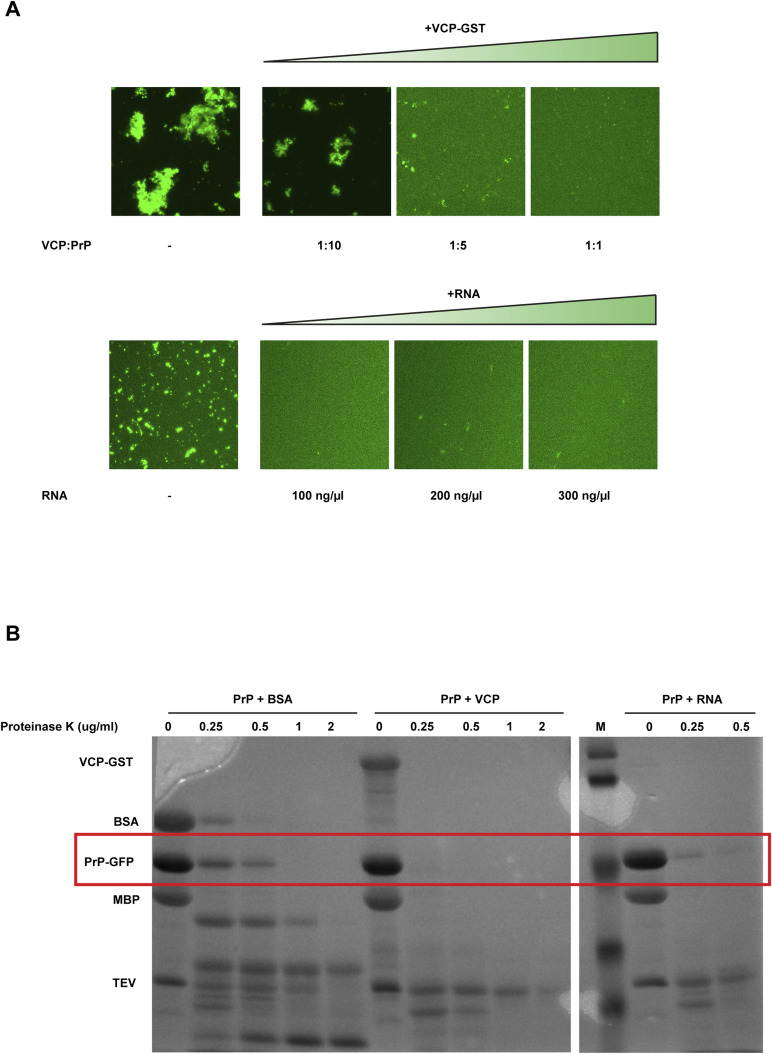
VCP/p97 and RNA inhibit aggregation of PrP-GFP in vitro. **(A)** 5 μM MBP-PrP-GFP was incubated in the presence of tobacco etch virus (TEV) protease for 1 h at RT in aggregation buffer (10 mM Tris–pH 7.4, 150 mM NaCl). To analyze aggregation of PrP-GFP fluorescence, imaging data were recorded by laser scanning microscopy using Z-stack and processed with maximum intensity projection. Upper row: increasing amounts of VCP-GST were added together with TEV. Lower row: increasing amounts of bulk RNA prepared from HeLa cells were added together with TEV. **(B)** VCP/p97 and RNA maintain PrP-GFP in a soluble conformation that is sensitive to proteinase digestion. 5 μM MBP-PrP-GFP was incubated in the presence of TEV protease for 1 h at RT in aggregation buffer (10 mM Tris–pH 7.4, 150 mM NaCl) in the presence of either BSA (5 μM), VCP-GST (5 μM), or bulk purified RNA from HeLa cells (300 ng/μl). The samples were treated with the indicated concentrations of proteinase K for 15 min at 37°C. The reaction was stopped by addition of 5 mM PMSF, and the samples were analyzed by SDS–PAGE and Coomassie brilliant blue staining.

What is the role of VCP/p97 in nuclear targeting of PrP? We hypothesized that VCP/p97 prevents PrP aggregation in the cytosol, thereby facilitating importin-β-mediated nuclear import. To study a possible anti-aggregation activity of VCP/p97, we established an in vitro aggregation assay with purified components. This assay is based on a recombinant MBP-PrP-GFP fusion protein. The N-terminal MBP (maltose-binding protein) keeps PrP soluble during purification and can be cleaved off by TEV (tobacco etch virus) protease to initiate phase transition of PrP (Fig 3F) (Kamps et al, 2021). When MBP is cleaved off, PrP-GFP is no longer soluble in physiological buffer (10 mM Tris–pH 7.4, 150 mM NaCl) and rapidly aggregates (Fig 3F, row 1). Aggregation of PrP-GFP after TEV cleavage was prevented in the presence of recombinant VCP/p97 (Fig 3F, row 2). Upon addition of ATP to the PrP-VCP/p97 complex, PrP-GFP formed aggregates, suggesting that the anti-aggregation activity of VCP/p97 was because of a specific interaction of VCP/p97 with PrP (Fig 3F, row 2). In line with this notion, the VCP/p97 inhibitor NMS-873, which inhibited nuclear import of PrP in cells (Fig S1C), also induced PrP-GFP aggregation by releasing PrP-GFP from its binding to VCP/p97 (Fig 3F, row 3). A titration analysis with increasing VCP/p97 concentrations showed that equimolar amounts of VCP/p97 are required to prevent aggregation of PrP (Fig S3A, upper panels).

To probe for conformational differences between VCP/p97-bound PrP-GFP and aggregated PrP-GFP, we performed limited proteolysis experiments. In complex with VCP/p97, PrP-GFP was more sensitive to proteolytic digestion compared to aggregated PrP (PrP + BSA) (Fig S3B). The in vitro approaches indicate that VCP/p97 acted as a holdase to prevent a conformational transition of soluble PrP-GFP into aggregates. These results also confirmed the cell culture data that the interaction of non-ER-imported PrP with VCP/p97 is independent of ubiquitination.

### PrP forms partially PK-resistant aggregates in the cytosol but not in the nucleus

**Figure 4.**
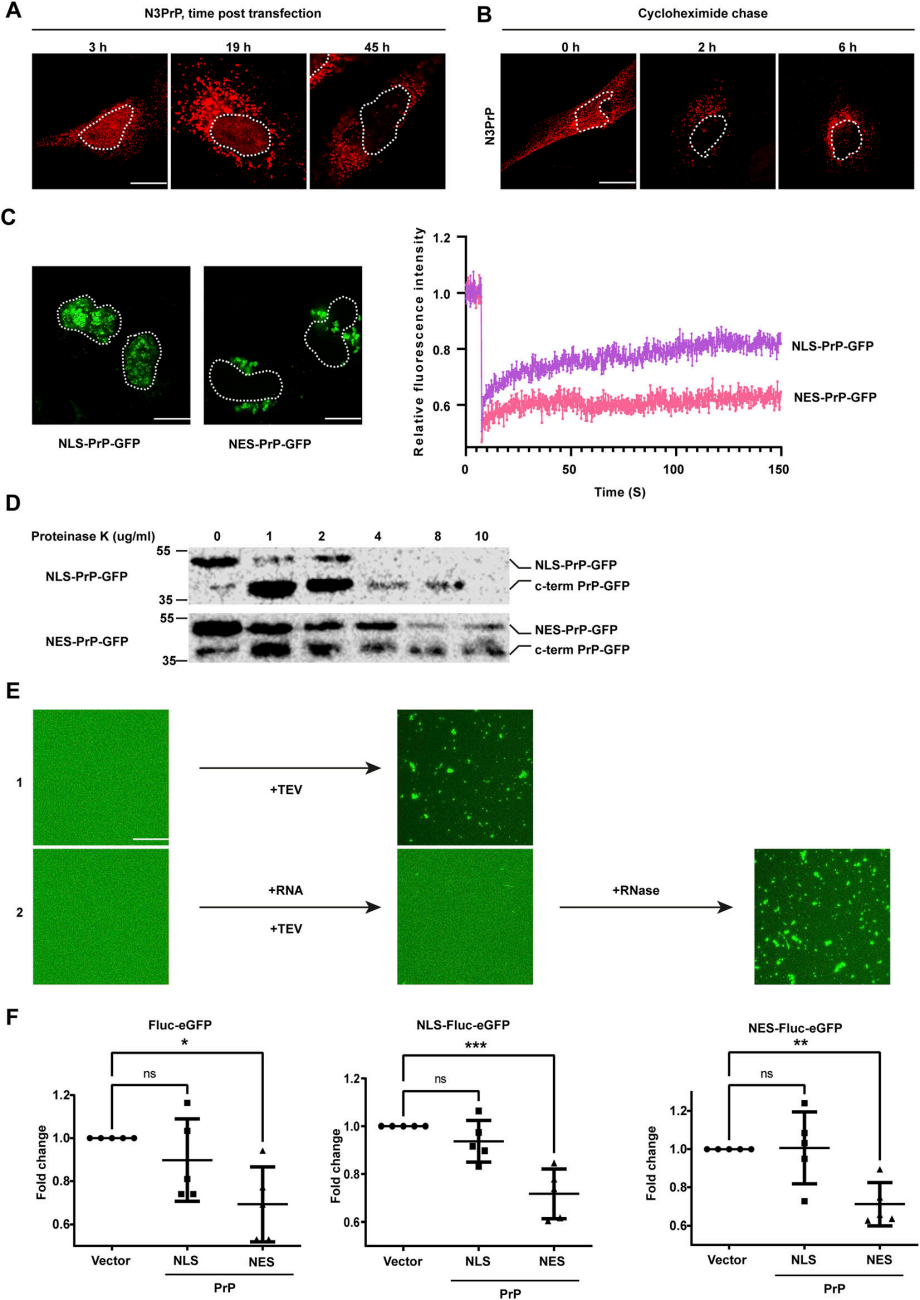
Cytosolic but not nuclear PrP forms partially protease-resistant aggregates and causes proteostasis decline. **(A)** Accumulation of cytosolic PrP interfere with nuclear translocation of PrP. SH-SY5Y cells were transiently transfected with N3PrP and cultured for the indicated time. Cells were then fixed, stained with antibodies against PrP and analyzed by SR-SIM. Dotted lines represent the nuclear boundary of the analyzed cells. **(B)** Nuclear PrP is degraded faster than cytosolic PrP. SH-SY5Y cells were transiently transfected with N3PrP. 24 h post-transfection, cells were treated for 2 or 6 h with 50 μg/ml cycloheximide to inhibit translation. Cells were then fixed, stained with antibodies against PrP, and analyzed by SR-SIM. Nuclei were stained with DAPI (merged). White dotted lines indicate boundary of the nucleus. **(C)** Nuclear PrP forms dynamic protein assemblies. SH-SY5Y cells were transiently transfected with NLS-PrP-GFP or NES-PrP-GFP. 24 h post-transfection, live cells were analyzed by FRAP to probe for the mobility of the PrP molecules. Relative fluorescence intensity was calculated and plotted against the time period of analysis. Total of seven cells were analyzed by FRAP for each condition, and mean relative fluorescence intensity is shown on the plot. White dotted lines indicate boundaries of the nuclei. **(D)** Cytosolic PrP forms partially proteinase-K-resistant aggregates. HEK293T cells were transfected with NLS-PrP-GFP or NES-PrP-GFP. 24 h post-transfection, cells were lysed and digested with different concentrations of proteinase K as indicated for 15 min at 37°C. Cell lysates were analyzed by immunoblotting using antibodies against PrP. The PrP antibody used (3F4) recognizes the amino acids 106–115, indicating that the faster migrating band represents truncated PrP-GFP molecules devoid of the N-terminally intrinsically disordered domain. **(E)** RNA inhibits aggregation of PrP-GFP in vitro. (1) 5 μM MBP-PrP-GFP was incubated in the presence of tobacco etch virus protease for 1 h at RT in aggregation buffer (10 mM Tris–pH 7.4, 150 mM NaCl). To analyze aggregation of PrP-GFP fluorescence, imaging data were recorded by laser scanning microscopy using Z-stack and processed with maximum intensity projection. (2) Bulk RNA prepared from HeLa cells was added together with tobacco etch virus. After 1 h the reaction was incubated for an additional 1 h in the presence of RNase. Aggregation was analyzed as in (1). **(F)** Cytosolic PrP, but not nuclear PrP, causes proteostasis decline in the cytosol and nucleus. Fluc-eGFP folding sensors present in both the nucleus and the cytosol (Fluc-eGFP), or only in the nucleus (NLS-Fluc-eGFP), or only in the cytosol (NES-Fluc-eGFP), were co-expressed in HEK cells with NLS-PrPΔGPI, or NES-PrPΔGPI. 24 h post-transfection, cells were lysed, and luminescence in total lysates was measured using a Luminometer. Fold change of luminescence was calculated by standardizing against lysates from cells transfected with an empty vector instead of PrP. The indicated line is the mean of the data set of five biological replicates, analyzed with Bonferroni’s multiple comparison test at 95% confidence interval, *P = 0.05, **P = 0.005, ***P = 0.0005. Scale bar, 10 μm.

During our study, we noticed that nuclear N3PrP decreased over time in contrast to cytosolic N3PrP (Fig 4A). An increase in cytosolic N3PrP was seen already at 19 h after transfection, whereas the nuclear fraction of N3PrP steadily decreased and was barely detectable at 45 h (Fig S2C, left panel). Moreover, cytosolic N3PrP formed detergent-insoluble aggregates (Fig S2C, right panel). To follow up on this observation, we analyzed the degradation of PrP in the cytosol and in the nucleus by treating cells transiently expressing N3PrP with cycloheximide (CHX) to inhibit protein translation. At the beginning of the CHX chase, N3PrP was detected both in the nucleus and in the cytosol. After 2 h of CHX treatment, the nuclear fraction of N3PrP almost disappeared, whereas N3PrP in the cytosol was still present 6 h after inhibiting protein translation (Figs 4B and S2D). The increased stability of N3PrP may indicate a misfolded conformation of N3PrP in the cytosol, which is resistant to degradation. To analyze possible differences in the biochemical and biophysical properties of PrP in the two different cellular compartments, we targeted PrP-GFP either to the nucleus or the cytosol by replacing the ER signal peptide with a NLS or nuclear export signal (NES). Fluorescence microscopy confirmed that NES-PrP-GFP and NLS-PrP-GFP are almost exclusively located in the cytosol and nucleus, respectively (Fig 4C, left panel). To probe for conformational differences, we recorded FRAP to quantify protein dynamics of NLS-PrP-GFP and NES-PrP-GFP in living cells. Fluorescence recovery of the NES-PrP-GFP assemblies in the cytosol was clearly reduced in comparison to that of the NLS-PrP-GFP assemblies, indicating differences in their material properties (Fig 4C, right panel).

As another approach to study conformational differences, we performed limited proteolysis experiments. Whole-cell lysates from NLS-PrP-GFP- or NES-PrP-GFP-expressing cells were treated with increasing concentrations of proteinase K (PK) before immunoblotting. At PK concentrations that completely digested NLS-PrP-GFP, the cytosolically localized NES-PrP-GFP resisted proteolytic degradation, suggesting that NES-PrP-GFP adopted a more aggregated conformation compared to NLS-PrP-GFP (Fig 4D). This analysis also revealed that the N-terminal domain of PrP is highly sensitive to proteolytic digestion, consistent with its intrinsically disordered structure.

### RNA prevents phase transition of PrP into aggregates

The FRAP recordings revealed that nuclear PrP is in a more soluble conformation compared to cytosolic PrP. A major difference between the cytosolic and nuclear chemical milieu is the high content of negatively charged RNAs in the nucleus, which can buffer protein aggregation (Maharana et al, 2018). To test for a possible role of RNA in keeping PrP-GFP soluble, we used the in vitro aggregation assay described above (Fig 3F). Indeed, bulk RNAs purified from HeLa cells interfered with the transition of soluble PrP-GFP into aggregates (Fig 4E, row 2, Fig S3A, lower panels). The anti-aggregation activity was dependent on RNA polymers since PrP-GFP started to aggregate upon addition of RNase (Fig 4E, row 2). Moreover, in the presence of RNAs, PrP-GFP was more sensitive to proteolytic digestion, indicating that RNA acted as negatively charged polymeric cosolute to keep PrP-GFP in a soluble conformation (Fig S3B).

### Cytosolic but not nuclear PrP causes a proteostasis decline

The FRAP and limited proteolysis experiments revealed that PrP adopts distinct conformations in the cytosol and the nucleus, respectively. We therefore wondered whether these conformational differences translate into specific physiological consequences. To monitor the cellular proteostasis capacity, we expressed a mutated version of the conformationally unstable firefly luciferase (Fluc) protein fused to eGFP. Proteotoxic stress leads to misfolding of Fluc-eGFP, resulting in decreased luciferase activity (Gupta et al, 2011; Park et al, 2013; Blumenstock et al, 2021). After co-expression of Fluc-eGFP with either NES- or NLS-PrP, the enzymatic activity of Fluc-eGFP was quantified by a luciferase assay. In cells expressing NLS-PrP, luciferase activity was not decreased compared to control cells. In contrast, a significant decline in luciferase activity was observed in cells expressing NES-PrP (Fig 4F, left panel). To specifically detect compartment-specific alterations in proteostasis, we targeted Fluc-eGFP either to the nucleus (NLS-Fluc-eGFP) or to the cytosol (NES-Fluc-eGFP). The co-expression of NLS-PrP did not affect the luciferase activity of neither cytosolic nor nuclear Fluc-eGFP. However, PrP accumulation in the cytosol upon NES-PrP expression significantly decreased the luciferase activity of both NLS-Fluc-eGFP and NES-Fluc-eGFP (Fig 4F, middle and right panels).

### Proteotoxic stress disrupts nuclear translocation of non-ER-imported PrP and induces the formation of self-perpetuating PrP aggregates in the cytosol

**Figure 5.**
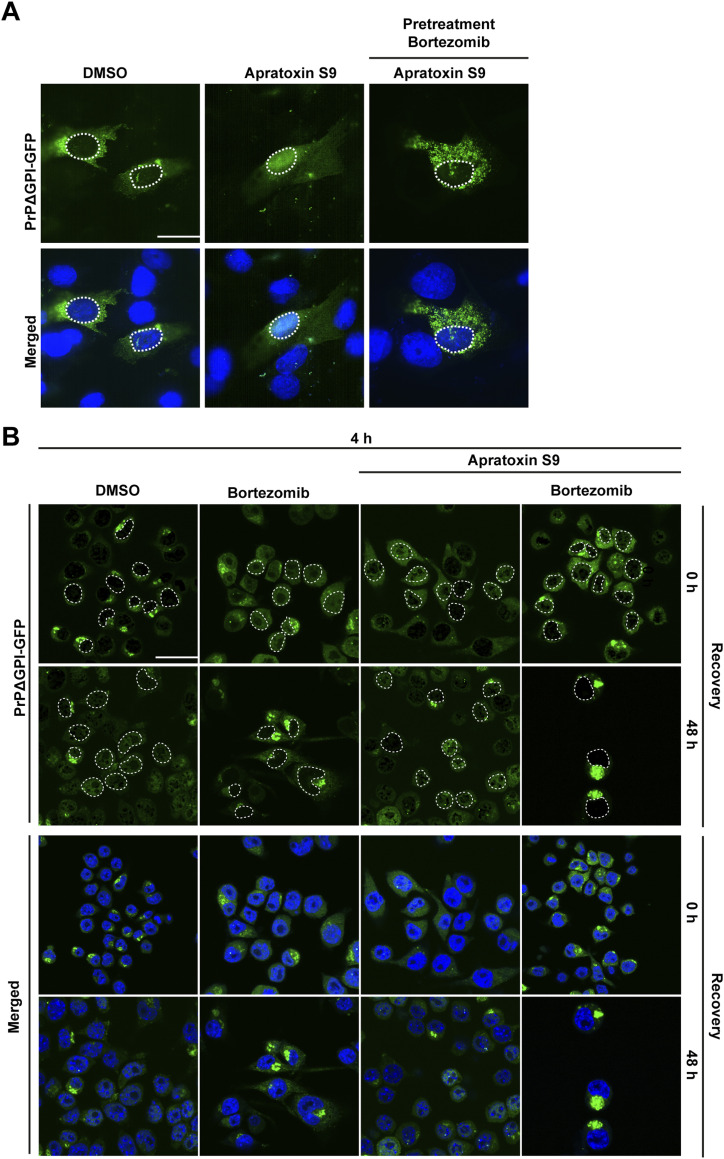
Cytosolic PrP forms self-perpetuating aggregates that disrupt further nuclear targeting of PrP. **(A)** Short-term proteasomal inhibition impairs subsequent nuclear targeting of non-ER-imported PrP. SH-SY5Y cells were transiently transfected with PrPΔGPI-GFP. 24 h post-transfection, cells were pre-treated for 3 h with Bortezomib (1 μM). The cells were washed and incubated for 4 h in fresh media containing Apratoxin S9 (100 nM). GFP fluorescence of fixed cells was analyzed by SR-SIM. White dotted lines indicate boundaries of the nuclei. Nuclei were stained with DAPI (merged). **(B)** PrP forms self-perpetuating aggregates in the cytosol that impair nuclear targeting of PrP. N2a cells stably expressing PrPΔGPI-GFP were plated and treated for 4 h with Apratoxin S9 (100 nM), and/or Bortezomib (0.5 μM) as indicated. **(B)** Cells were lysed either immediately (0 h recovery) or washed with fresh medium and cultured for additional 48 h (48 h recovery). The cells were fixed and GFP fluorescence was analyzed by SR-SIM either directly (0 h recovery) or washed with fresh medium and cultured for additional 48 h (48 h recovery). White dotted lines indicate boundaries of the nuclei. Nuclei were stained with DAPI (merged). Scale bar, 10 μm.

**Figure S4.**
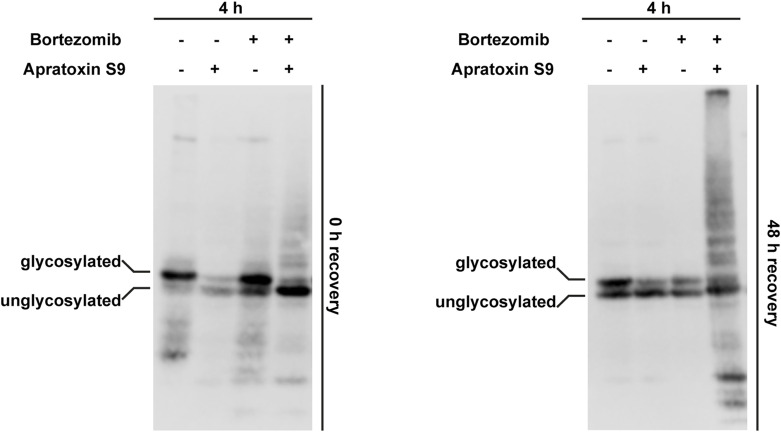
PrP forms self-perpetuating aggregates in the cytosol that can recruit newly synthesized PrP. N2a cells stably expressing PrPΔGPI-GFP were plated and treated for 4 h with Apratoxin S9 (100 nM) and/or Bortezomib (0.5 μM) as indicated. Cells were lysed either immediately (0 h recovery) or washed with fresh medium and cultured for additional 48 h (48 h recovery). Cell lysates were analyzed by immunoblotting using antibodies against PrP.

As shown above, cytosolic PrP aggregates increased over time upon defective ER import, whereas the relative amount of nuclear PrP decreased (Fig 4A and B). This observation led us to hypothesize that the cytosolic PrP aggregates and proteotoxic stress induced by these aggregates disrupt further nuclear import of newly synthesized PrP. To test this possibility experimentally, we induced proteotoxic stress in cells expressing PrPΔGPI-GFP by transiently inhibiting the proteasome with Bortezomib (3 h pretreatment). Then, the reversible proteasome inhibitor was washed out, and ER import was inhibited by Apratoxin S9. After additional 4 h, the cells were fixed and analyzed by fluorescence microscopy. In cells treated with Apratoxin S9 only, the typical nuclear localization of non-ER-imported PrPΔGPI-GFP was observed. However, pretreatment with Bortezomib interfered with the transport of PrPΔGPI-GFP into the nucleus and induced the formation of cytosolic PrP aggregates (Fig 5A). To address possible long-term effects of transient proteotoxic stress, we induced the formation of cytosolic PrPΔGPI-GFP aggregates by Bortezomib and Apratoxin S9 (4 h) and analyzed the cells either directly or after 48 h recovery in fresh medium by Western blotting and fluorescence microscopy. The Western blot analysis of cells after 4 h Apratoxin S9 treatment revealed the typical loss of glycosylated PrP and the appearance of unglycosylated, non-ER-imported species (Fig S4). This was accompanied by the targeting of PrP to the nucleus (Fig 5B). In cells treated with both Apratoxin S9 and Bortezomib for 4 h, the non-ER-imported PrP was not targeted to the nucleus but remained in the cytosol (Fig 5B). The corresponding Western blot analysis confirmed that PrP was not imported into the ER under these conditions since the glycosylated PrP species disappeared (Fig S4).

After recovery for 48 h in fresh medium, nuclear PrP mostly disappeared from cells that had been pre-treated with Apratoxin S9 alone (Fig 5B). However, large perinuclear PrP aggregates were present in the cytosol of cells 48 h after exposure to Apratoxin S9 and the proteasomal inhibitor Bortezomib for 4 h (Fig 5B). These PrP aggregates were formed from non-ER-imported PrPΔGPI-GFP, permanently generated under physiological conditions because of the inefficient ER signal peptide of PrP (Kim et al, 2001; Rutkowski et al, 2001; Drisaldi et al, 2003; Heller et al, 2003; Heske et al, 2004; Rane et al, 2004; Hegde & Bernstein, 2006; Rambold et al, 2006; Miesbauer et al, 2009). In sum, these experiments indicated that short-term proteotoxic stress leads to the formation of self-perpetuating PrP aggregates in the cytosol that interfere with further nuclear targeting of non-ER-imported PrP.

## Discussion

VCP/p97 plays a central role in the ERAD pathway by targeting non-native secretory proteins to cytosolic proteasomes for degradation. Here, we identified a novel role of VCP/p97 in counteracting proteotoxic stress induced by mislocalized secretory proteins. VCP/p97 can promote nuclear targeting of non-ER-imported aggregation-prone clients in a ubiquitination-independent manner.

**Figure 6.**
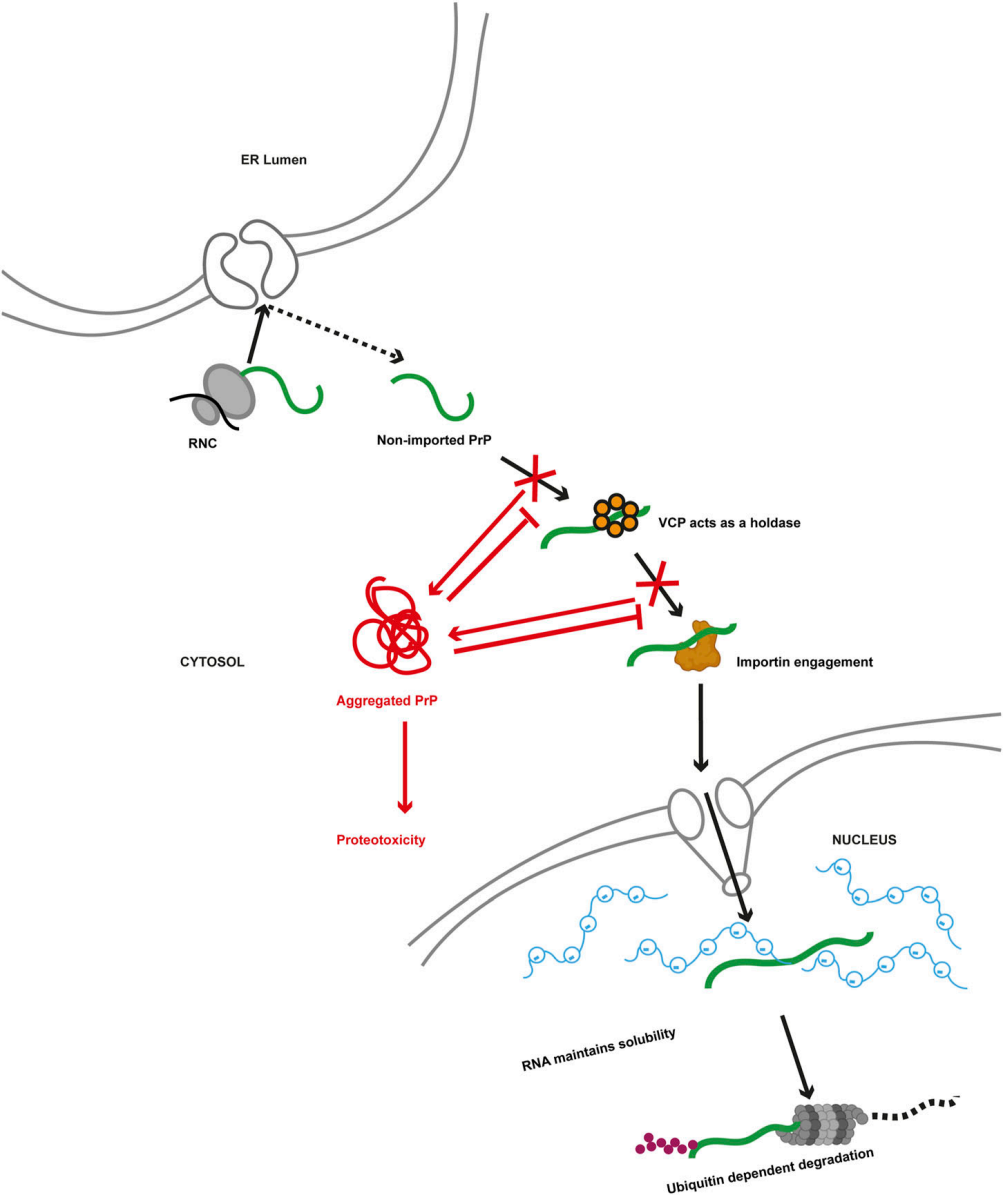
Schematic summary of the findings. Under physiological conditions, a sequential interaction of non-ER-imported PrP with VCP/p97 and importins maintains PrP in a soluble conformation in the cytosol and mediates its import into the nucleus. High concentrations of RNA buffer aggregation of PrP in the nuclear compartment and facilitate the ubiquitin-dependent proteasomal degradation of PrP. Under proteotoxic stress conditions, the interactions with VCP/p97 and importins are disturbed, and PrP forms self-perpetuating cytosolic aggregates that prevent further nuclear targeting of non-ER-imported PrP.

PrP is an ideal model protein to study the consequences of non-ER-imported species since, even under physiological conditions, a significant fraction of PrP is not imported into the ER, based on its inefficient ER signal peptide and the presence of a large intrinsically disordered N-terminal domain (Kim et al, 2001; Rutkowski et al, 2001; Drisaldi et al, 2003; Heller et al, 2003; Heske et al, 2004; Rane et al, 2004; Hegde & Bernstein, 2006; Rambold et al, 2006; Miesbauer et al, 2009). Whereas the molecular determinants underlying the impaired ER import of PrP have been studied in great detail, little is known about the fate of non-ER imported PrP. PrP has been reported to accumulate in the cytosol of cells treated with proteasomal inhibitors (Ma & Lindquist, 2001, 2002; Yedidia et al, 2001; Drisaldi et al, 2003). Therefore, previous studies concluded that after failed ER import, PrP is targeted to cytosolic proteasomes for degradation, similar to “classical” ERAD substrates. However, our study revealed a different, more complex pathway. Using a variety of novel in vitro and in cellulo approaches, we observed that non-ER-imported PrP is targeted to the nucleus and provided insights into the underlying mechanism (Fig 6). Similar to ERAD substrates, PrP interacted with VCP/p97; however, this interaction did not result in targeting PrP to cytosolic proteasomes for degradation. Instead, VCP/p97 was required to maintain non-ER-imported PrP in a soluble conformation after the release of PrP from ribosomes. Notably, this activity was independent of VCP/p97 adaptor proteins and PrP ubiquitination. Purified VCP/p97 prevented the aggregation of non-ubiquitinated PrP in vitro in the absence of VCP/p97 adaptor proteins, indicating that VCP/p97 acts as a holdase (Thoms, 2002). Moreover, an inhibitor of E1 ubiquitin-activating enzymes did not interfere with binding of PrP to VCP/p97 and nuclear targeting of PrP in cellular models. However, the flexible N-terminal tail of VCP/p97 was required for the interaction with non-ER-imported PrP, corroborating previous findings that this domain of VCP/p97 can mediate an interaction with non-ubiquitinated substrates (van den Boom et al, 2023).

The in vitro experiments also revealed that VCP/p97 does not prevent aggregation of PrP in the presence of ATP. Thus, in a cellular context, PrP only transiently binds to VCP/p97, and a subsequent interaction with importin-ß and import into the nucleus is required to prevent aggregation of PrP in the cytosol. Indeed, the interaction of PrP with VCP/p97 was a prerequisite for nuclear targeting of PrP by importin-ß: VCP/p97 inhibitors induced aggregation of PrP in the cytosol and prevented the importin-ß-mediated nuclear translocation of PrP. The subcellular localization of VCP/p97 in close proximity to ER membranes allows its rapid binding to PrP upon failed ER import and release from the Sec61 translocon. Similarly, inhibiting binding of PrP to importin-ß triggered cytosolic aggregation of PrP, adding to the notion that importins have chaperone-like activity (Guo et al, 2018; Hofweber et al, 2018; Qamar et al, 2018; Fare et al, 2023).

Surprisingly, non-ER-imported PrP was not efficiently degraded by cytosolic proteasomes, although it interacted with VCP/p97. Possibly, the cytosolic VCP/p97-PrP complex is not associated with VCP/p97 cofactors required for targeting clients to the proteasome. Alternatively, the conformation of PrP plays a role. Our FRAP recordings and limited proteolysis experiments revealed that cytosolic and nuclear PrP assemblies were different regarding their material properties: nuclear PrP was in a soluble conformation and mobile, whereas cytosolic PrP formed partially PK-resistant, immobile aggregates. How can these compartment-specific PrP conformations be explained mechanistically? One relevant difference between the cytoplasm and nucleoplasm is the increased abundance of RNAs in the nucleus. Our in vitro experiments indicated that RNAs prevent the aggregation of PrP, suggesting that RNAs can act as negatively charged polymeric cosolutes to keep PrP in a soluble conformation.

Finally, our study suggested that impaired nuclear translocation of cytosolic PrP and its aggregation in the cytosol are pathophysiologically relevant. In contrast to nuclear PrP, cytosolic PrP assemblies caused an imbalance in proteostasis. Moreover, cytosolic PrP aggregates formed after transient proteasomal inhibition self-perpetuated and interfered with nuclear targeting of newly generated non-ER-imported PrP, even when proteasomal function was restored. The toxicity of cytosolic PrP aggregates is emphasized by a recent study in which we showed that cross-seeding by cytosolic PrP inactivates TDP-43 (Polido et al, 2024). In a wider perspective, our study highlights the concept that mislocalization of proteins to non-native cellular compartments can trigger their aggregation with adverse effects on proteostasis balance (Hartl, 2017; Yanagitani et al, 2017; Juszkiewicz & Hegde, 2018). Interestingly, the nucleus has recently been identified as a destination for the proteasomal degradation of non-imported mitochondrial precursors (Shakya et al, 2021), suggesting that the nucleus is a more general quality control compartment for proteins mislocalized in the cytosol. It will now be interesting to characterize both shared and specific key players in alleviating proteotoxic stress in the cytosol by targeting aggregation-prone proteins to the nucleus.

## Materials and Methods

### DNA constructs and siRNA

Plasmid maintenance and amplification were carried out using *Escherichia coli* TOP10© (Thermo Fisher Scientific). All PrP constructs were generated by standard PCR cloning techniques and are based on the coding region of mouse PRNP (GenBank accession number M18070) modified to express PrP-L108M/V111M, allowing detection by the monoclonal antibody 3F4. All the mammalian expression plasmids were cloned into pcDNA3.1(+)-Neo vector (V79020; Invitrogen). PrPΔGPIGFP: aa 1–226 tagged with GFP at the C-terminus; PrPΔGPI: aa 1–226; NES-PrP-GFP: aa 1–226 tagged with GFP at the C-terminus and with a nuclear export signal at the N-terminus; NLS-PrP-GFP: aa 1–226 tagged with GFP at the C-terminus and with a NLS at the N-terminus; NES-PrP: aa 1–226 tagged with a nuclear export signal at the N-terminus; NLS-PrP: aa 1–226 tagged with a NLS at the N-terminus; N3PrP: mouse PrP with a mutated ER signal peptide (Kim et al, 2002); PrPΔNLSΔGPI: mouse PrP aa 1–226 with K23A, K24A, R25A, and K27A substitutions were cloned into pcDNA3.1(+)-Neo via HindIII and XhoI. The modified signaling sequences are as follows:

NLS: 5′ ATGCCACCAAAAAAAAAAAGAAAAGTT 3′

NES: 5′ ATGCTGGAACTTCTGGAGGATTTGACACTG 3′

N3 signal peptide: 5′ATGGCGAACCTTGGCGATGACCTGCTGGCCCTCTTTGTGACTATGTGGACTGATGTCGGCCTCTGC 3′

Generation of pMAL-MBP-TEV-PrP-eGFP-TEV-His_6_ is described elsewhere (Kamps et al, 2021). pcDNA5FRT/TO-p97-EQ-mycStrep, pcDNA5FRT/TO-p97wt-mycStrep, and pGEX-6P-1-p97 were kindly provided by Hemmo Meyer (Ritz et al, 2011). Fluc-eGFP, NLS-Fluc-eGFP, and NES-Fluc-eGFP were kindly provided by F. Ulrich Hartl and Irina Dudanova (Gupta et al, 2011; Park et al, 2013; Blumenstock et al, 2021).

pcDNA3.1(+)-Neo was used to subclone VCP mutant lacking aa 1–207 (Δ1-207 TrapVCP) and with a strep tag at C-terminus via NheI and BamHI, VCP mutant was amplified from pcDNA5FRT/TO-p97-EQ-mycStrep using following primers:

Forward: 5′ ATATGCTAGCATGTGCAGGAAGCAGCTAGCTC 3′

Reverse: 5′ ATATGGATCCTTTTTCGAACTGCGGGTGG 3′

The following siRNA was used to knock down BAG6 and RNF126: (ambion Silencer Select pre-designed).

RNF126: 5′ GCAUCUUCGAUGACAGAGCUUTT 3′ (siRNA ID: s31185).

BAG6: 5′CAAGAGCAGUUUAAUAGCATT 3′ (siRNA ID: s15468).

### Cell culture and transfection

#### HEK293T, HeLa cells, MEFs

Cells were cultured in DMEM supplemented with 10% (vol/vol) FBS, 100 IU/ml penicillin, and 100 μg/ml streptomycin sulphate.

#### Human neuroblastoma SH-SY5Y cells

Cultured in DMEM F-12 (DMEM/F12) supplemented with 15% (vol/vol) FBS, 100 IU/ml penicillin, 100 μg/ml streptomycin sulphate, and 1 x MEM non-essential amino acid solution (Gibco).

#### Mouse neuroblastoma N2a cells

Cells were cultured in MEM supplemented with 10% (vol/vol) FBS, 100 IU/ml penicillin, and 100 μg/ml streptomycin sulphate. N2a stable cell lines were maintained in 0.1% puromycin (Santa Cruz, Germany). N2a stably expressing PrPΔGPIGFP was established by using pHulk plasmid system (Haase et al, 2010).

All cell lines were grown in humidified conditions at 37°C with 5% CO_2_ and passaged when cells reached 80% confluence. For inhibitor treatment, cells were incubated in complete growth medium with the indicated drug at 37°C. Unless described otherwise, transfections were performed using the following procedures:

For SH-SY5Y and MEF cells, Lipofectamine and Plus Reagent (Invitrogen) and, for HeLa cells, Lipofectamine 2000 (Invitrogen) were used according to the manufacturer’s instructions. HEK293T cells were transfected using PEI (polyethylenimine). DNA and PEI were mixed in a 1:4 ratio (μg:μl) in Opti-MEM and incubated for 10 min at RT. The DNA-PEI mixture was then added to the cells.

### Primary neuron culture

Primary neurons were prepared following the protocol from Life Technologies, with modifications. All media and solutions were sterile filtered using 0.22 μm membrane filter. Cortices were explanted from embryos 16–18 DPC (day post coitum), carefully washed thrice with ice cold Dissection Buffer (9.9 mM HEPES, 137 mM sodium chloride, 5.4 mM potassium chloride, 0.17 mM sodium phosphate dibasic anhydrous, 0.22 mM potassium phosphate monobasic anhydrous, 33.3 mM D-glucose, 43.8 mM sucrose, pH 7.4), and treated with 1 mg acetyl trypsin for 10 min at 37°C in the water bath. Cortices were then rinsed three times in dissection buffer, and 100 U DNase-I was added. Complete dissociation was performed manually using a series of increasingly smaller pipette tips, followed by straining through a 70 μm filter (EASYSTRAINER, Greiner Bio One) to remove larger tissue residues and diluted with neurobasal medium (with 10% FBS, and 0.5 mM glutamine). The number of cells was determined using a haemocytometer, and the neurons were plated on poly-L-lysine-coated plates at densities of 1.3 × 10^4^ and 1.3 × 10^6^ cells/well in 24-well plates and 60-mm dishes, respectively. The medium was changed to neurobasal medium supplemented with B-27 supplement and 0.5 mM glutamine after 50 min. The primary cortical neurons were cultured in a humidified incubator at 37°C with 5% CO_2_. Five DIV (day in vitro) neurons were transfected using Lipofectamine 2000 following the manufacturer’s protocol and analyzed for immunofluorescence after 48 h. Animal protocols were performed in compliance with institutional and governmental regulations.

### Immunofluorescence and confocal microscopy

Transfected cells were washed with PBS (Gibco) and then fixed using 4% (wt/vol) PFA for 10 min. Cells were permeabilized using 0.2% Triton X-100 in PBS for 10 min and washed with PBS. For blocking, cells were incubated for 1 h in 5% normal goat serum in PBS and incubated in 0.1–0.2% (vol/vol) primary antibody in the blocking solution overnight at 4°C. Cells were then washed with PBS and incubated with the corresponding 0.1% (vol/vol) secondary antibody in PBS for 1 h at 25°C. The secondary antibody was removed and washed away using PBS and 0.2% Tween 20 in PBS for 5 min. All coverslips were incubated in 0.1% (vol/vol) DAPI in Millipore, washed with Millipore water, and then mounted using Fluoromount-G (Invitrogen). For non-permeabilized cells, after fixation, coverslips were counterstained with DAPI and mounted.

Fluorescence images were acquired using the ELYRA PS.1 microscope equipped with an LSM880 (Carl Zeiss) and a 20x, 63x oil or 100x oil immersion objective. Super-resolution images were generated by structured illumination microscopy (SR-SIM) using 405, 488, and 561 nm widefield laser illumination. SIM confocal images were processed using the raw scale mode of ZEN2.3 software (Carl Zeiss). In detail, for each channel, five phase images in three different rotations were acquired. After SIM processing, the confocal section thickness (z-scaling) is either 0.126 μm (AlexaFluor555 and DAPI) or 0.110 μm (AlexaFluor488/GFP and DAPI) based on the optical properties of the Zeiss Plan-Apochromat 63x/NA1,4 Oil DIC M27 objective in combination with the 405, 488, and 561 nm diode laser lines. For the quantification of nuclear fluorescence intensities, confocal images were acquired with the imaging settings kept uniform among replicates.

### Immunoblotting

Proteins were fractionated by SDS–PAGE and transferred to nitrocellulose or polyvinylidene difluoride membranes by electroblotting. The nitrocellulose membranes were blocked with 5% non-fat dry milk or 5% BSA in TBST (TBS containing 0.1% Tween 20) for 60 min at RT and subsequently incubated with the primary antibody diluted in blocking buffer for 16 h at 4°C. After extensive washing with TBST, the membranes were incubated with horseradish peroxidase-conjugated secondary antibody for 60 min at RT. After washing with TBST, the antigen was detected with the ECL detection system (Promega) as specified by the manufacturer with Azure Sapphire Biomolecular Imager (Azure Biosystems).

### Biochemical analyses

#### Preparation of whole cell lysates and detergent-soluble/insoluble fractions

As described previously (Tatzelt et al, 1996), cells were washed twice with cold PBS, scraped off the plate, and pelleted by centrifugation. For whole cell lysates, the cells were lysed in 2X Laemmli sample buffer. To separate detergent-soluble and -insoluble fractions, the cells were lysed in cold detergent buffer (0.5% Triton X-100 and 0.5% sodium deoxycholate [DOC] in PBS) and centrifuged at 15,000*g* for 20 min at 4°C before the Western blot analysis in Laemmli sample buffer. Supernatants and pellets were examined by immunoblotting.

### PNGase-F digestion and glycosylation assessment

In a six-well plate, HEK293T cells were seeded, grown overnight, and transfected with PrPΔGPI-GFP. After 16 h, the cells were treated with or without 100 nM Apratoxin S9 for 4 h. The cells were harvested (1,000*g*, 5 min), lysed in 1% Triton X-100 in PBS, vortexed, and incubated on ice for 10 min. The lysates were centrifuged at 18,000*g* for 10 min, and the resulting supernatants were treated with PNGase-F (New England Biolabs) for 3 h at 37°C. The samples were analyzed through sodium dodecyl sulphate polyacrylamide gel electrophoresis (SDS–PAGE) using a 10% SDS–PAGE gel and immunoblotted using an antibody, 3F4, against PrP.

### Co-immunoprecipitation

In 6-cm dishes, HEK293T cells were seeded and co-transfected with PrPΔGPI and p97Wt-mycStrep or p97-EQ-mycStrep. 24 h post-transfection, cells were harvested (1,000*g*, 5 min), lysed with 500 μl lysis buffer (150 mM KCl, 5 mM MgCl_2_, 50 mM Tris–HCl pH 7.4, 1% Triton X-100, 5% glycerol, 2 mM β-mercaptoethanol supplemented with Complete EDTA-free protease inhibitors and PhosSTOP, Roche), gently resuspended by pipetting, and incubated on ice for 20 min. The lysates were centrifuged at 18,000*g* for 15 min at 4°C, and the supernatant (input) was collected in separate tubes. MagStrep “type3” XT beads (IBA Lifesciences GmbH) pre-washed with lysis buffer were added to the supernatants and incubated for 2 h at 4°C with gentle rotation. The flowthrough was removed, and the beads were washed thrice with 500 μl lysis buffer. Finally, the beads were boiled with 50 μl 2x Laemmli sample buffer for 5 min to elute the immunocomplex. All samples were fractionated by SDS–PAGE gels and further analyzed through immunoblotting against VCP and PrP.

### Luciferase assay

HEK293T cells were seeded and co-transfected with Fluc-GFP(Wt)/NLS-Fluc-GFP(Wt)/NES-Fluc-GFP(Wt) and NLS-PrPΔGPI/NES-PrPΔGPI/pcDNA3.1(+) empty vector control. 24 h post-transfection, cells were harvested (1,000*g*, 5 min), lysed in 100 μl 1X reporter lysis buffer (Promega), vortexed, and centrifuged (17,049*g*, 4°C, 15 min). The resulting supernatant (20 μl) was added into a 96-well plate in quadruplicate. Before loading the plate into the Cytation5 reader (BioTek), the injectors were washed following the manufacturer’s protocol and primed with the luciferase assay substrate (Promega) diluted in water (1:10). 100 μl of the substrate was injected per well, and the luminescence was measured at 557 nm. The empty vector controls were used to normalize all the samples within the same transfection group and plotted to calculate the fold change in luminescence of the Fluc reporter.

### FRAP analyses

SH-SY5Y cells were seeded on a 35-mm IBIDI μ-Dish and transfected with NLS-PrP-GFP or NES-PrP-GFP. 24 h post-transfection, cells were imaged in Invitrogen Live Cell Imaging Solution. ZEN2.1 bleaching and region software module and Plan-Apochromat 100× numerical aperture 1.46-oil differential interference contrast M27 objective was used for imaging the cells. For each cell analyzed, two circular regions of interest were chosen. One region was bleached with 100% laser power and a pixel dwell time of 8.71 ms, with a scan time of 111.29 ms, and the other region was used as the reference signal. Relative fluorescence intensity (RFI) was calculated at time t using the following equation: RFI = I_BL_(t)/I′_BL_/I_Ref_(t)/I′_Ref_, where I_BL_(t) and I_Ref_(t) are the intensities measured at time t in the photobleached region and the reference region, respectively. I′_BL_ and I′_Ref_ are the intensities measured before photobleaching.

### Proteinase K digestion

HEK293T cells were seeded and transfected with NLS-PrP-GFP or NES-PrP-GFP. 24 h post-transfection, cells were lysed in 200 μl lysis buffer (1% Triton X-100 in 50 mM HEPES pH 7.4, 150 mM NaCl) on ice for 15 min. The lysate was cleared at 17,000*g* for 10 min, and equal amounts of the supernatant (20 μl) were digested with the indicated concentrations of proteinase K for 15 min at 25°C. The reaction was stopped with 5 mM PMSF (phenylmethylsulfonyl fluoride), mixed with Laemmli sample buffer, and boiled for 1 min. Samples were analyzed by immunoblotting against a PrP antibody specific to C-terminus (POM-15) and GFP.

### Expression and purification of recombinant proteins

Plasmid maintenance, bacterial expression, and purification of MBP-PrP-GFP were performed as described earlier (Kamps et al, 2021). For expression and purification of GST-tagged VCP, transformed *E. coli* BL21 (DE3) were grown to an OD 600 of 0.6–0.8 before induction with 0.5 mM isopropyl-β-d-thiogalactopyranoside and a change in temperature to 18°C overnight. Cells were harvested and resuspended in lysis buffer (50 mM HEPES pH 8.0, 150 mM KCl, 2 mM MgCl_2_, 5% glycerol) with 20 ml/liter of culture volume, protease inhibitor cocktail, and 1 mg/ml of Lysozyme added to the suspension, stirred with a stir-bar gently for 30 min at 4°C, and lysed by a French press. After centrifugation for 60 min at 20,000*g* at 4°C, the lysate was cleared with a 0.8 µm filter. GST-tagged VCP/p97 was affinity-purified with a GSTTrap FF column (GE Healthcare) with a flow rate of 1–5 ml/min on an ÄKTA purification system. The column was washed with the lysis buffer and the bound proteins were eluted using the lysis buffer but with 20 mM glutathione. Adding glutathione changes the pH; buffer needs to be adjusted to pH7.4–8.0 before use. Eluted fractions were assessed for protein content by SDS–PAGE. Desired fractions were pooled, concentrated to <5 ml for injection from loop onto gel filtration column, and further purified using HiLoad 16/600 Superdex 200 pg at 1 ml/min flowrate with gel filtration buffer (50 mM HEPES pH 7.4, 150 mM KCl, 2 mM MgCl_2_, 5% glycerol, 1 mM DTT). Eluted fractions were checked for protein content by SDS–PAGE. Desired fractions were pooled, concentrated to the desired concentration, flash frozen in liquid nitrogen, aliquoted, and stored at −80°C. The protein concentration was determined using the absorbance at 280 nm and the extinction coefficient of each protein by NanoDrop 2000 (Thermo Fisher Scientific).

### Sample preparation for in vitro aggregation assay

Protein samples were thawed and centrifuged (20,000*g*, 10 min, 4°C) to remove aggregates. Afterwards, the buffer was exchanged to 10 mM Tris–pH 7.4 using Vivaspin 500 columns with 30,000 D cut off (Sartorius Stedim Biotech). The samples were centrifuged (12,000*g*, 7 min, 4°C) five times for complete buffer exchange, and then the protein concentration was determined by NanoDrop 2000. To induce aggregation of PrP-GFP, the buffer was supplemented with 150 mM NaCl and incubated with TEV protease for 1 h before microscopy.

### Limited proteolysis of recombinant PrP-GFP

5 μM MBP-PrP-GFP in aggregation buffer was cleaved with TEV protease in the presence of BSA, GST-VCP, or bulk RNA prepared from HeLa cells. The samples were treated for 15 min at 25°C with increasing concentrations of proteinase K as indicated. After stopping the reactions with 5 mM PMSF (phenylmethylsulfonyl fluoride), the samples were analyzed by SDS–PAGE and Coomassie brilliant blue staining.

### Quantification and statistical analysis

CellProfiler (https://cellprofiler.org) was used to calculate ratio of nuclear to cytosolic mean GFP intensity of each cell, by using DAPI as a marker for nuclear mask. The macro used for this analysis was “Human C-N translocation” written for automated image analysis which is available on the CellProfiler website. The in vitro samples were analyzed as described previously (Ahlers et al, 2021; Kamps et al, 2021). Statistical analyses for the luciferase assays and nuclear GFP intensity quantification were performed using one-way ANOVA (Kruskal-Wallis test with Dunn’s post-test at 95% confidence interval, * = *P* < 0.05) and *t* test (Mann-Whitney test, two-tailed at 95% confidence interval, * = *P* < 0.05), respectively, using the GraphPad PRISM 9.5 software.

## Supplementary Material

Reviewer comments
